# Spatial and Temporal Variation of Lead, Cadmium, and Zinc in Feathers of Great Tit and Blue Tit Nestlings in Central Poland

**DOI:** 10.1007/s00244-014-0028-4

**Published:** 2014-05-07

**Authors:** Marcin Markowski, Mirosława Bańbura, Adam Kaliński, Janusz Markowski, Joanna Skwarska, Jarosław Wawrzyniak, Piotr Zieliński, Jerzy Bańbura

**Affiliations:** 1 Department of Experimental Zoology and Evolutionary Biology, Faculty of Biology and Environmental Protection, University of Łódź, Banacha 12/16, 90-237 Lodz, Poland; 2 Department of Teacher Training and Biological Diversity Studies, Faculty of Biology and Environmental Protection, University of Łódź, Banacha 1/3, 90-237 Lodz, Poland; 3 Natural History Museum, Faculty of Biology and Environmental Protection, University of Łódź, Kilińskiego 101, 90-237 Lodz, Poland; 4 Department of Ecology and Vertebrate Zoology, Faculty of Biology and Environmental Protection, University of Łódź, Banacha 12/16, 90-237 Lodz, Poland

## Abstract

In this study, we examined heavy-metal concentrations in feathers of nestling great tits *Parus major* and blue tits *Cyanistes caeruleus* at two different sites (urban parkland *vs*. deciduous forest) located in the Łódź agglomeration in relation to interyear variation. We found that tit species did not differ significantly in lead and cadmium concentrations. Zinc concentration was significantly higher in blue tits. We also found that lead and cadmium levels in blue tit nestlings and the level of lead in great tit nestlings were higher in the parkland site than in the woodland site. We explain habitat variation in heavy-metal concentrations in feathers of nestlings by different levels of contamination at study sites. For both tit species, significant variation in heavy-metal amounts accumulated by nestlings was found between years with the lowest value in a year with the lowest value of rainfall. We suggest that the interyear variation may be accounted for by differences in rainfall, thus influencing quantities of trace elements bioavailable in the environment.

Urbanization and industrial processes exert a great influence on environment, mostly by contributing large amounts of toxic trace elements. Therefore, an important task is to identify reliable methods to evaluate and monitor heavy-metal pollution. Living organisms provide useful data on the bioavailability and toxicity of metals and are good bioindicators (Burger 1993; Dauwe et al. 2002; Swaileh and Sansur 2006). The use of various species classified as bioindicators allows us to evaluate heavy-metal impacts on different types of environment. Furthermore, contamination level may be monitored in time, thus giving an idea of past, current, and future condition of the ecosystem in which the organism lives. In addition, using organisms to monitor the presence of heavy metals in the environment provides accurate information on how they may affect physiological and behavioral processes (Holt and Miller 2011).

Studies of heavy-metal concentration in birds are performed on various organs and products, for example, liver, kidney, and muscle tissues, bones, fat, eggs, feathers, and excrement (Burger 1993; Dauwe et al. 2000; Linder and Grillitsch 2000). Because determining metal loads in internal organs involves the necessity to kill the studied individuals, the use of feathers, excrement, and blood samples should be treated as a noninvasive method (Burger 1993; Dauwe et al. 2000, 2002; Scheifler et al. 2006). Particularly feathers can be easily sampled without affecting the health and condition of individual birds. Moreover, feather samples do not require any special storage conditions, thus facilitating sampling in the field (Burger 1993; Dmowski 1999).

Some passerines, such as the great tit *Parus major* and blue tit *Cyanistes caeruleus*, fulfill the criteria of indicator organisms. They are numerous, ubiquitous, high-positioned in the food chain, and well-studied species (Lack 1955; Perrins 1965; Eeva and Lehikoinen 1996; Dauwe et al. 2002). Moreover, both great and blue tits readily use man-made nest boxes; thus, their breeding populations can be easily established to monitor and study in a particular area of interest (Eens et al. 1999; Dauwe et al. 2002; Janssens et al. 2003). Both species are resident and forage in relatively small territories (Cramp and Perrins 1993). Using nestlings as biomonitors has some advantages. Because tit nestlings reside inside the nest boxes during their development, the main type of exposure to heavy metals is by way of food supplied by parents (Furness 1993). Therefore, studying heavy metals in nestlings may help to identify and evaluate local heavy-metal pollution (Burger and Gochfeld 1994; Janssens et al. 2003; Berglund et al. 2011). Sampling nestlings provides data on possible heavy-metal accumulation during the limited period of nestling growth during which the evident toxicological effects may be observed (Furness 1993; Dauwe et al. 2000).

Great tits and blue tits turned out to be useful in monitoring heavy-metal contamination in different studies using both adults (Eens et al. 1999; Dauwe et al. 2002; Zou et al. 2005; Deng et al. 2007) and nestlings (Dauwe et al. 2000; Dauwe et al. 2004a; Janssens et al. 2002; Eeva et al. 2009). Different effects of heavy-metal pollution have been found: reproductive parameters (Dauwe et al. 2005; Eeva et al. 2009), size and thickness of egg shells (Eeva and Lehikoinen 1995; Dauwe et al. 2004b), condition and health of nestlings (Eeva and Lehikoinen 1996; Dauwe et al. 2003), and morphometric and hematological parameters (Eeva et al. 2000, 2009; Snoeijs et al. 2004; Geens et al. 2009). In some studies, the investigators emphasized ambiguous results concerning the effects of heavy-metal pollution on morphological and physiological parameters (Eeva et al. 1997; Dauwe et al. 2006).

Although both great and blue tits occur sympatrically, they may vary in their vulnerability to metal accumulation. This may be related to differences in trophic niches, diet, and metabolic rates (Burger and Gochfeld 2009; Eeva et al. 2009; Berglund et al. 2011). Few studies exist that analyze and compare heavy-metal concentrations between these two species (Eens et al. 1999; Dauwe et al. 2002). Surprisingly, studies comparing the accumulation of metals by great and blue tit nestlings are rare, particularly those using feathers. Only Dauwe et al. (2000) has performed such an analysis. There are also few studies concerning long-term variation in heavy-metal pollution (Dmowski 1997; Berglund et al. 2010). It seems that it should be an aspect of particular interest to biomonitoring studies to evaluate pollution during different periods of time because levels of pollutants can change rapidly.

The purpose of this study was to analyze the variation in heavy-metal concentrations in feathers of nestling great tits and blue tits inhabiting two different sites located in the Łódź with respect to interyear source of variation. The city of Łódź was characterized in the past by a dynamic development of industry, which strongly influenced the local environment (Wesołowski 2002). Currently despite the changes in the economic structure and the collapse of many major manufacturing plants, the city is still among most polluted urban areas in Poland. Increasing traffic and considerable number of households with heating systems without any special devices to reduce pollutants originating from combustion processes have a significant contribution to the emission of pollutants, particularly heavy metals (Bem et al. 2003). Therefore, we chose to analyze lead, cadmium, and zinc because they are toxic metals presumed to occur and remain active in the study area.

## Materials and methods

The study was performed during the breeding seasons of 2006–2008 as part of a long-term project on the breeding biology of tits around Łódź in central Poland (Bańbura et al. 2007). All procedures were approved by the local Ethical Committee and the State Office for Environment Protection.

### Study Sites

Study sites were located within administrative boundaries of Łódź in two differing habitats: urban parkland and deciduous forest. The parkland area is situated in the southwest part of Łódź and includes botanical and zoological gardens covering approximately 80 ha (Kaliński et al. 2009). This area is situated approximately 3 km from the city center and is surrounded by roads of considerable traffic intensity, thus contributing one of the highest line emissions in Łódź ranging from 4.47 to 7.74 mg/y (Raport o Stanie Środowiska 2009). The parkland site is characterized by a mosaic, highly fragmented arrangement of vegetation and a visible influence of human activity connected with urban processes. The forest site covers approximately 140 ha in the central part of the Łagiewniki Forest (total of 1,250 ha). Tree stands consist of mature deciduous trees of diverse species with predomination of oaks *Quercus robur* and *Q.* *petraea* (Marciniak et al. 2007). This area is situated in the northeast part of Łódź, further away from the city center, compared with the parkland site. The forest complex in which the study site was established has a low number of roads, which are to the most extent excluded from traffic. Consequently, the Łagiewniki Forest area is characterized by a decreased influence of human activity and car traffic. Study sites were separated by a distance of 10 km (Kaliński et al. 2009).

### Sample Collection

The study sites were supplied with wooden nest-boxes (approximately 180 in the parkland area and 250 in the forest). Regular inspections of nest-boxes started at the beginning of April in each breeding season. The purpose of inspections was to record nesting bird species; basic breeding traits, such as laying date, clutch size, and brood size at the moment of hatching; and number of fledglings. The nestlings were individually ringed and measured (wing length and body mass) when they were 13–15 days old (Bańbura et al. 2007). At the same time, two proximal secondary flight feathers were sampled from each nestling from the same brood to avoid the effect of differences in nestling development. Feathers taken from the nestlings of the same brood were pooled into one sample. They were kept at −18 °C until the time of laboratory analyses. In total, we evaluated heavy metals in 219 samples collected during the breeding seasons 2006–2008, including 123 samples from great tits (61 from parkland and 62 from forest site) and 96 from blue tits (23 from parkland and 73 from forest site). More detailed description of sample size, including species, year, and study site, is listed in the tables herein.

At the end of May 2008, we also collected 10 vegetation samples: 6 from the urban parkland area (*Tilia cordata, Q.* *rubra, Carpinus betulus* leaves) and 4 from the forest site (*C.* *betulus, Q.* *robur* leaves). The leaves were of average size, undamaged, and taken from a height of 2–3 m at ≤10 m away from nest boxes (Dauwe et al. 2004a).

### Heavy-Metal Analysis in Samples

The feathers used for analysis were rinsed in deionized water to remove externally adsorbed heavy metals (Pilastro et al. 1993; Pain et al. 2005; Metcheva et al. 2006). The samples were dried for 24 h in the oven at 60 °C. The leaf samples were not washed so that surface deposition could be estimated as well (Dauwe et al. 2004a). They were dried in an oven at 65 °C for 72 h and then weighed and ground into a powder (Fayiga et al. 2007). Subsequently, feather and leaf samples were digested in a 4:1 mixture of 65 % nitrogenous acid and 70 % perchloric acid. After the end of the digestion process, all samples were diluted in deionized water and stored in polypropylene metal-free vials at −18 °C until analysis (Markowski et al. 2013).

The analyses were performed in the Laboratory of Computer and Analytic Techniques, Faculty of Biology and Environmental Protection, University of Łódź. Lead, cadmium, and zinc concentrations were measured in the samples using atomic absorption spectrophotometer Spectr AA-300. Lead and cadmium, due to very their low concentrations in samples, were measured using electro-thermal atomizer GTA96. Zinc was determined in the flame atomizer using the technique of low-temperature plasma. In one case, lead concentration was not detected; thus, it was omitted from the statistical analysis. The detection limit for lead was 0.02 ng/g, for cadmium was 0.006 ng/g, and for zinc was 8 ng/g. The samples were analyzed in batches with the use of blind samples and determination of calibration curves. All metal concentrations were expressed in µg/g on a dry-weight basis. We used certified reference material (ERM-CE278 k) from the Institute for Reference Materials and Measurements (Geel, Belgium) to verify the quality and accuracy of the performed analysis. Recovery rates for the certified reference material were within an acceptable margin.

### Analysis of Rainfall

Data on rainfall for Łódź during the study seasons were obtained from http://www.tutiempo.net/en/Climate/Lodz (Tutiempo Network, S.L., Madrid, Spain), a Web site containing records provided by the state weather station located in Łódź. We calculated individual total rainfall for every brood as a sum of daily rainfall during the period starting on March 1 and ending 20 days after the hatch of a given brood. Those sums were used as indicators of rainfall intensity, thus indirectly influencing the level of pollutants in the food of particular broods. For both great and blue tits, we assumed that fledglings leave the nest on the day 20 day after hatch (Gibb 1950; Gosler 1993).

### Statistical Analysis

Statistical analyses of data were performed using Statistica 9.1 software (StatSoft, Inc. 2010). All statistical analyses were performed on ln-transformed values of response variables to normalize their distributions and stabilize variances (Sokal and Rohlf 1981). Arithmetic means of nontransformed heavy-metal concentrations per nest with SE values are presented in tables and figures.

To analyze the overall differences in heavy-metal concentrations between great and blue tit nestlings from two different study sites during 3 consecutive years, multivariate analysis of variance (MANOVA) was used. Heavy-metal concentrations were used as a multiple dependent variable with species, site, and year as factors. We applied the protected ANOVA approach by examining concentrations of particular metals using univariate ANOVAs after obtaining significant effects in MANOVA, which led to assessing which individual dependent variables contributed to significant multivariate effects (Scheiner 1993). To examine differences among years, we used Tukey’s honestly significant difference (HSD) test preceded by Fisher LSD test. A similar approach to MANOVA, as an introductory step to protected univariate ANOVA, was also used to test differences in heavy-metal concentrations between years within the same site and between sites within each year for each study species separately. To evaluate differences in heavy-metal concentrations in vegetation samples from the two study areas, MANOVA followed by univariate ANOVA was used as well. To analyze the differences in rainfall between breeding seasons, we used ANOVA. Correlations between metals were calculated as Pearson product-moment correlation coefficients. In all statistical analyses, we assumed a level of significance at *p* < 0.05 (Sokal and Rohlf 1981).

## Results

### Heavy-Metal Correlations

All considered heavy-metal concentrations were intercorrelated with a tendency to slightly higher correlations for great tits compared with blue tits (Table 1). The consequence of this result is the application of MANOVA as an introductory step in the further analyses.

**Table 1:** Pearson product-moment correlation coefficients for lead, cadmium, and zinc concentrations in feathers of great tit (*n* = 123, above diagonal) and blue tit nestlings (*n* = 95, below diagonal)

	Lead	Cadmium	Zinc
Lead	–	0.62*	0.68*
Cadmium	0.45*	–	0.46*
Zinc	0.55*	0.43*	–

* *p* < 0.05

### Interspecific Variations in Heavy-Metal Concentrations

MANOVA showed that blue tits differed from great tits in concentrations of one or more included metals in nestling feathers (Table 2). Univariate ANOVA for each metal showed that there was no significant species effect for lead (univariate ANOVA: *F*_1,206_ = 0.01 *p* = 0.92) or cadmium (univariate ANOVA: *F*_1,206_ = 1.84, *p* = 0.18) concentration in feathers of nestlings, although great tits tended to show slightly higher values (Table 3). In contrast, zinc concentration was significantly higher (univariate ANOVA: *F*_1,206_ = 15.81 *p* < 0.0001) in feathers of blue tit than in great tit nestlings (Table 3).

**Table 2:** Results of MANOVA for total characteristics of mean concentrations of lead, cadmium, and zinc as a multiple dependent variable in relation to species as the main factor with site and year as background factors

Effect	Wilk’s lambda	*F*	*df*	Error *df*	*p*
Species	0.87	10.1	3	204	<0.0001
Site	0.91	7.12	3	204	0.0001
Year	0.69	13.92	6	408	<0.0001
Species*site	0.98	1.44	3	204	0.23
Species*year	0.95	1.63	6	408	0.14
Site*year	0.88	4.41	6	408	0.0002
Species*site*year	0.99	0.49	6	408	0.82

*df* degrees of freedom

**Table 3:** Metal concentrations in feathers of great and blue tit nestlings^a^

	Great tit (*n*)	Blue tit (*n*)
Lead	4.28 ± 0.59 (123)	3.14 ± 0.40 (95)
Cadmium	0.65 ± 0.16 (123)	0.29 ± 0.04 (96)
Zinc	150.86 ± 6.70 (123)	200.60 ± 7.97 (96)

^a^Given are mean ± SE in µg/g on a dry-weight basis

Sample sizes are given in parentheses

### Intraspecific Variations in Heavy-Metal Concentrations

MANOVAs were further performed for multiple dependent variables characterizing metal concentrations separately in great tit and blue tit with respect to two study sites and 3 consecutive years of study. We did not find a significant differences between the urban parkland and forest areas in the case of great tit nestlings (Table 4).

**Table 4:** Results of MANOVA for total characteristics of mean concentrations of lead, cadmium, and zinc as multiple dependent variables characterizing feathers of great and blue tit nestlings in relation to site and year as a factors

Effect	Wilk’s lambda	*F*	*df*	Error *df*	*p*
Site					
Great tit	0.97	1.32	3	115	0.27
Blue tit	0.77	8.54	3	87	<0.0001
Year					
Great tit	0.70	7.42	6	230	<0.0001
Blue tit	0.57	9.50	6	174	<0.0001
Site*year					
Great tit	0.84	3.36	6	230	0.003
Blue tit	0.89	1.80	6	174	0.1

For this species, no intersite difference was shown in univariate ANOVA for lead (univariate ANOVA: *F*_1,117_ = 3.24, *p* = 0.07), cadmium (univariate ANOVA: *F*_1,117_ = 2.71, *p* = 0.1), and zinc (univariate ANOVA: *F*_1,117_ = 0.84, *p* = 0.36) (Table 5). In contrast, MANOVA showed that there were differences between the study sites in the case of blue tit nestlings (Table 4), which resulted from significant differences in lead (univariate ANOVA: *F*_1,89_ = 17.72, *p* < 0.0001) and cadmium (univariate ANOVA: *F*_1,90_ = 5.97, *p* = 0.01) concentrations (Table 5). The concentrations of lead and cadmium were higher at the urban parkland than at the forest site (Table 5). In contrast, zinc values were higher in the forest area; however, comparison of study sites did not yield a significant result (univariate ANOVA: *F*_1,90_ = 1.25, *p* = 0.27) (Table 5).

**Table 5:** Metal concentrations in feathers of great and blue tit nestlings from two habitats in relation to year variation

	Parkland site (*n*)				Forest site (*n*)			
	2006	2007	2008	2006–2008	2006	2007	2008	2006–2008
Great tit								
Lead	7.93 ± 2.68	1.46 ± 0.19	8.85 ± 2.05	4.41 ± 0.84	3.82 ± 1.42	6.33 ± 2.87	3.15 ± 0.31	4.14 ± 0.84
	(5)	(36)	(20)	(61)	(16)	(16)	(30)	(62)
Cadmium	2.35 ± 1.49	0.08 ± 0.01	0.58 ± 0.07	0.43 ± 0.14	0.23 ± 0.06	1.99 ± 1.12	0.59 ± 0.09	0.86 ± 0.30
	(5)	(36)	(20)	(61)	(16)	(16)	(30)	(62)
Zinc	229.76 ± 92.54	115.11 ± 7.17	187.40 ± 9.77	148.20 ± 10.13	171.21 ± 16.77	137.70 ± 28.64	152.45 ± 5.24	153.49 ± 5.24
	(5)	(36)	(20)	(61)	(16)	(16)	(30)	(62)
Blue tit								
Lead	5.72 ± 2.74	1.54 ± 0.26	16.83 ± 1.46	4.92 ± 1.31	1.98 ± 0.54	1.78 ± 0.71	3.46 ± 0.32	2.57 ± 0.29
	(4)	(15)	(4)	(23)	(25)	(16)	(31)	(72)
Cadmium	0.51 ± 0.25	0.10 ± 0.03	1.14 ± 0.75	0.35 ± 0.15	0.17 ± 0.05	0.14 ± 0.04	0.42 ± 0.07	0.27 ± 0.04
	(4)	(15)	(4)	(23)	(25)	(17)	(31)	(73)
Zinc	226.11 ± 14.89	157.59 ± 11.85	263.59 ± 19.16	187.94 ± 12.50	238.54 ± 21.19	175.94 ± 22.74	192.92 ± 6.46	204.59 ± 9.71
	(4)	(15)	(4)	(23)	(25)	(17)	(31)	(73)

Given are mean ± SE in ppm (µg/g) on a dry-weight basis

Sample sizes are given in parentheses

Differences among years in overall metal concentrations were significant for both great tit and blue tit nestlings (Table 4). Univariate ANOVA performed for each species showed that all studied elements varied significantly between years. Subsequently, results of post hoc comparisons by applying Tukey’s HSD test showed significant differences among each study year (Table 6). Fisher’s LSD test, which preceded Tukey’s test, provided similar results except for recording significant differences for cadmium concentrations measured in feathers of great tit nestlings between 2006 and 2007.

**Table 6:** Metal concentrations in feathers of great and blue tit nestlings in subsequent years (pooled data from parkland and forest site)

Species	2006	2007	2008	*p* ^a^
Great tit				
Lead	4.80 ± 1.28A (21)	2.96 ± 0.93B (52)	5.43 ± 0.92A (50)	<0.0001
Cadmium	0.74 ± 0.39A (21)	0.67 ± 0.36A (52)	0.58 ± 0.06B (50)	<0.0001
Zinc	185.13 ± 24.49A (21)	122.06 ± 10.04B (52)	166.43 ± 5.53A (50)	<0.0001
Blue tit				
Lead	2.50 ± 0.62A (29)	1.66 ± 0.38A (31)	4.99 ± 0.79B (35)	<0.0001
Cadmium	0.22 ± 0.06A (29)	0.12 ± 0.03A (32)	0.51 ± 0.10B (35)	0.0001
Zinc	236.83 ± 18.32A (29)	167.34 ± 13.20B (32)	201.00 ± 7.16A (35)	0.0005

^a^ *p* values from univariate ANOVA applied to characterize differences in lead, cadmium, and zinc concentrations in feathers of great and blue tit nestlings among years of study. Differences among years of study using Tukey’s HSD test are shown by letters “A” and “B.” Means with the same letter indicate a nonsignificant test result. Given are mean ± SE in ppm (µg/g) on a dry-weight basis

Sample sizes are given in parentheses

In MANOVA tests, we found a significant interaction between study site and year only for great tit nestlings (Table 4). Univariate ANOVA showed that this resulted from a site-year interaction in cadmium concentration (univariate ANOVA: *F*_2,117_ = 9.01, *p* = 0.0002).

MANOVAs for multiple dependent variables characterizing metal concentrations were also applied to consider the differences between years within the same site for each species. We found that great tit nestlings differed among years of study at the parkland site (Wilk’s lambda: *F*_6,112_ = 12.85, *p* < 0.0001) and at the forest site (Wilk’s lambda: *F*_6,114_ = 3.36, *p* = 0.004). Univariate ANOVA showed that these resulted from significant differences in lead (univariate ANOVA: *F*_2,58_ = 18.73, *p* < 0.0001), cadmium (univariate ANOVA: *F*_2,58_ = 44.67, *p* < 0.0001), and zinc (univariate ANOVA: *F*_2,58_ = 11.47, *p* < 0.0001) concentrations in the case of the parkland site and cadmium concentrations (univariate ANOVA*: F*_*2*,58_ = 5.72, *p* = 0.005) at the forest site. At the parkland site, we noted a characteristic pattern of all heavy-metal concentrations with the lowest values being recorded in 2007. In contrast, cadmium levels at the forest site were lowest in the first study year (Table 5).

Similarly, for blue tit nestlings, MANOVA showed differences among study seasons at the parkland site (Wilk’s lambda: *F*_6,36_ = 6.45, *p* = 0.0001) and at the forest site (Wilk’s lambda: *F*_6,134_ = 9.62, *p* < 0.0001). Respectively, this resulted from significant differences in lead (univariate ANOVA: *F*_2,20_ = 20.38, *p* < 0.0001), cadmium (univariate ANOVA: *F*_2,20_ = 13.00, *p* = 0.0002), and zinc (univariate ANOVA: *F*_2,20_ = 6.87, *p* = 0.005) concentrations at the parkland site as well as lead (univariate ANOVA: *F*_2,69_ = 11.97, *p* < 0.0001), cadmium (univariate ANOVA: *F*_2,69_ = 6.32, *p* = 0.03), and zinc (univariate ANOVA: *F*_*2*,69_ = 5.86, *p* = 0.004) concentrations at the forest site. At both study sites, the lowest values of all considered trace elements were recorded in 2007 (Table 5).

Next we tested differences between sites within each year for both tit species separately by applying MANOVA for multiple dependent variables characterizing metal concentrations. For great tit nestlings, we did not find significant differences among study sites in 2006 (Wilk’s lambda: *F*_3,17_ = 2.54, *p* = 0.09) and 2007 (Wilk’s lambda: *F*_3,48_ = 2.31, *p* = 0.09). However, in the last year of study the differences were significant (Wilk’s lambda: *F*_3,46_ = 4.20, *p* = 0.01), which resulted from significantly higher lead (univariate ANOVA: *F*_1,48_ = 8.57 *p* = 0.005) and zinc (univariate ANOVA: *F*_1,48_ = 10.66, *p* = 0.002) concentrations at the parkland site (Table 5).

In the case of blue tit nestlings, MANOVA test showed differences between study sites in 2006 (Wilk’s lambda: *F*_3,25_ = 5.26, *p* = 0.006) and 2008 (Wilk’s lambda: *F*_3,31_ = 11.97, *p* < 0.0001). It was a result of significant differences in lead (univariate ANOVA: *F*_1,27_ = 4.93, *p* = 0.03) and cadmium (univariate ANOVA: *F*_1,27_ = 4.7, *p* = 0.03) concentrations in 2006 and lead (univariate ANOVA: *F*_1,33_ = 38.07, *p* < 0.0001), and zinc (univariate ANOVA: *F*_1,33_ = 11.14, *p* = 0.002) concentrations in 2008. Higher concentrations of these heavy metals were noted at the parkland site (Table 5).

### Intersite Variation of Heavy-Metal Concentrations in Vegetation Samples

MANOVA performed for the multiple dependent variable composed of the concentrations of lead, cadmium and zinc in vegetation samples, with study site as a factor, showed that the parkland area differed from the forest in the metal concentrations measured in leaves (Wilk’s lambda: *F*_3,6_ = 39.08, *p* = 0.0002). Univariate ANOVA showed that all studied elements varied significantly between sites (Table 7), reaching higher concentrations of lead and cadmium in leaves of trees collected at the parkland site, whereas zinc concentration was higher at the forest site (Table 7).

**Table 7:** Comparison between metal concentrations in leaves collected from two study sites

Metal	Parkland site (*n*)	Forest site (*n*/*F/df/p*)
Lead	23.95 ± 7.53 (6)	5.56 ± 1.53 (4)/6.85/1;8/0.03
Cadmium	5.13 ± 1.31 (6)	0.92 ± 0.50 (4)/11.05/1;8/0.01
Zinc	36.11 ± 2.18 (6)	58.26 ± 8.34 (4)/11.33/1;8/0.009

Given are mean ± SE expressed in µg/g on a dry weight basis

Sample sizes are given in parentheses

### Interannual Variation of Rainfalls

**Fig. 1:**
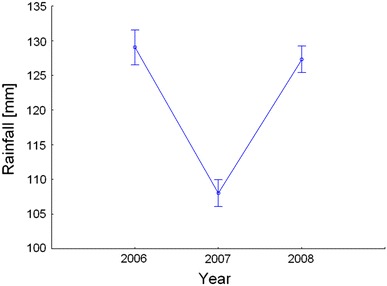
Mean per-brood sum of rainfall (±SE) in 2006–2008 with totals of rainfall from March 1 until day 20 after hatch for each studied brood

Weather-data analysis showed significant differences between years (one-way ANOVA: *F*_2,213_ = 55.5, *p* < 0.0001) considering the mean of sum of rainfall in each of the 3 study years. The first and last year was characterized by higher and comparable mean values of rainfall, whereas in 2007 we noted markedly lower precipitation (Fig. 1).

## Discussion

Relatively high positive correlations between heavy metals in feathers of great and blue tit nestlings found in this study are probably the result of co-occurrence of these elements in the environment. Orłowski et al. (2012), analyzing interactions of concentrations of trace elements in various organs and tissues of nestling rooks *C.* *fragilegus*, showed similar relationships. Some investigators have also suggested that a similar way of gathering food can yield such a result (Pinowski et al. 1983). Many studies on trace-element interactions in birds have presented antagonistic relations of lead and cadmium with zinc (Kamiński 1998; Dauwe et al. 2002; Kamiński and Warot 2005a, b). A lack of such antagonistic effects in our study is in agreement with results of Dauwe et al. (2002) concerning adult great and blue tits where high positive correlations between lead, cadmium and zinc were found in feathers.

Although, in general, mean lead and cadmium concentrations in feathers of great tit nestlings were higher than those in blue tits, the differences were nonsignificant. In contrast, zinc concentration was significantly higher in nestling blue tits than in great tits.

We are aware of only one other study performed on great and blue tit nestlings in which the investigators analyzed differences in the level of heavy-metal accumulation using feathers (Dauwe et al. 2000). These investigators showed that there were no significant interspecific differences in measured elements between great and blue tit. Similarly to our results, Dauwe et al. (2000) noted slightly higher concentrations of lead and cadmium for great tit and, in the case of zinc, higher values in feathers of blue tit. In this study, investigators found that both species accumulated much higher amounts of zinc than lead and cadmium. These results were also comparable with our findings concerning differences in amounts of lead, cadmium, and zinc accumulated in feathers of tit nestlings. Studies comparing the quantity of accumulated metals in the case of great and blue tit nestlings were also performed by Eeva et al. (2009). However in this study, trace elements were examined in nestling excrement without zinc evaluation for possible comparisons. The investigators showed that the levels of studied metals were relatively similar for both species, although there were some slight differences in levels of lead and cadmium with the values being higher for blue tit nestlings.

Many investigators have suggested that prey insects may be a main source of metal contamination accumulated by insectivorous birds, particularly nestlings (Nyholm et al. 1995; Eeva and Lehikoinen 1996; Berglund et al. 2011; and many others); consequently, variation in metal concentrations between blue tit and great tit nestlings is likely to be associated with an interspecies difference in the diet. Both of these tit species are known to prefer caterpillars as the main food for nestlings (Cholewa and Wesołowski 2011), but the blue tit forages almost exclusively in deciduous tree canopies, whereas the great tit is much more versatile and often forages in bushes and even on the ground (Lack 1971). An important addition to caterpillars, the principal food for great and blue tit nestlings are spiders (Török 1986; Cholewa and Wesołowski 2011; Michalski et al. 2011). Blue tits are more specialized in caterpillars, especially in small-sized individuals (Nour et al. 1998; Bańbura et al. 1999; Naef-Daenzer and Keller 1999; Török and Tóth 1999). In the case of spiders, the great tit preys mainly on spiders that live on the ground (Lycosoids), whereas blue tits choose spider species living on plants (Török 1986). Ground-dwelling spiders are recognized as being strong metal accumulators (Jung and Lee 2012). In addition, according to Eeva and Lehikoinen (1996), heavy metals might accumulate more in ground-living prey items. Therefore, differences in diet and foraging methods may have some effect on the interspecies variation in metal concentrations found in our study. In contrast, lower zinc concentration recorded for great tit nestlings may be explained by the physiological effects of pollution. According to Di Giulio and Scanlon (1985), increased levels of cadmium can enhance zinc concentrations in the kidneys, probably by inducing metallothionein. This could cause a decrease in zinc accumulation in feathers. Berglund et al. (2011), studying species-related differences between pied flycatcher and great tit nestlings, presented similar conclusions. They found a higher element concentration in feces and liver tissue of pied flycatcher *Ficedula hypoleuca* nestlings and explained this result by a large proportion of insects of higher trophic levels in the food of pied flycatchers (Eeva et al. 1997, 2005). The same conclusions come from the study of Eeva and Lehikoinen (1996) study also performed on pied flycatcher and great tit.

Some investigators explain variations in heavy-metal concentrations between species by differences in metabolic rate. Deng et al. (2007) found that smaller birds having higher metabolic rate showed faster accumulation of trace metals. Lebedeva (1997), comparing 24 bird species varying considerably in body size, also showed that smaller specimens accumulated more lead in bones than those with higher body mass. According to the investigator, this resulted from differences in metabolic rate as well. In contrast, our results do not support this explanation. We considered site and year of study as the main factors significantly affecting heavy-metal variations in nestling feathers of great and blue tit. Regarding the differences in foraging methods and type of food, as mentioned previously, we believe that it would be difficult and inappropriate to explain variations in heavy-metal amounts accumulated by both tit species simply relying only on metabolic rate. In addition, Dauwe et al. (2000) concluded that small differences in heavy-metal concentrations between great and blue tit nestlings cannot be entirely explained by differences in their metabolic rates.

The Łódź agglomeration has been recognized as an area of ecological threat in Poland, which was confirmed by a special directive of the Polish Government in 1983 (Krok 2004). This is explained by the fact that on a relatively small area (400 km^2^) with dense population, there are a few large power plants and many smaller local plants with inadequate fly-ash removal systems, which pollute local environment (Bem et al. 2003). Jankiewicz et al. (2000, 2001) found high heavy-metal accumulation in soil collected from allotment gardens near major roads and suggested that vehicle traffic is the main source of soil pollution in Łódź. In agreement with our expectations and the results of other investigators, our parkland study site was more contaminated than the forest site. We recorded 4 times higher lead and 5 times higher cadmium concentrations in vegetation samples collected from the parkland site compared with the forest site, although the latter one indicated 2 times higher zinc levels (Table 7). This can be explained by the fact that the parkland site is surrounded by roads with heavy traffic, which constitutes an important source of particulate matter (PM10) contributing heavy metals such as lead, cadmium and zinc (Apeagyei et al. 2011). Based on data from 2002 to 2008, it was recorded that PM10 concentrations exceeded the permissible level in Łódź city center and its vicinity (Raport o Stanie Środowiska 2009). Thus, the parkland site in our study system is located in a zone of higher contamination than the forest area considering lead and cadmium. The impact of traffic on heavy-metal accumulation was confirmed by Alfani et al. (2000), who clearly showed that concentrations of heavy metals in unwashed leaves of *Q.* *ilex* were several times higher along roads compared with urban and suburban parks in Naples. In contrast, higher values of zinc noted at the forest site may be related to the emission of combustion products from an EC3 power plant located in the north part of Łódź. According to Jankiewicz and Adamczyk (2010) this facility contributed to an increase of zinc in soil samples collected eastward from the power plant site. The investigators found that 26 % of samples collected east of the EC3 plant exceeded permissible levels of zinc. Considering that in Łódź, winds from the west and southwest directions are dominant, it can be assumed that this can be a relevant factor explaining increased levels of zinc at the forest site.

Our analysis of heavy-metal concentrations in feathers of tit nestlings collected between 2006 and 2008 showed differences between the study sites. In feathers of nestling blue tits, we found significantly higher lead and cadmium concentrations than in tits from the parkland site. In contrast, great tit nestlings did not differ between study sites, although nonsignificantly higher amounts of lead were recorded at the parkland site as well (Table 5). Such results may suggest that the blue tit may be considered a more sensitive species than the great tit, thus better allowing to identify and evaluate subtle differences in heavy-metal contamination. To confirm this conclusion, once again one should refer to interspecific dietary differences. According to Eeva et al. (2009), despite similarities in diet between great and blue tit, the latter is characterized by a higher quantum of aphids (Hemiptera) in food supplied to nestlings. This group of arthropods tends to be particularly abundant in polluted environments (Eeva et al. 2009) and as such we chose the parkland site in our study. Referring to an earlier study performed by Marciniak et al. (2007), our study sites varied in percentage share of aphid, with considerably higher numbers observed at the parkland site. Therefore, these facts can explain significant differences found for blue tits and nonsignificant ones for great tits. Consequently, we suggest that blue tit nestling in our study system will better reflect the possible level of contamination.

Considering the data from the year 2008 only, we found significantly higher levels of lead and zinc in feathers of both tit species from the parkland area. Concentrations of cadmium were comparable among study sites, as in the case of feathers sampled from great tit nestlings, and for blue tit we noted higher values at the parkland site although this result was nonsignificant. The results of analyses of feathers collected in 2008 to some extent correspond with the results obtained in the leaf samples analysis, which showed the parkland area as being a more polluted study site (Table 5).

A similar relation between heavy-metal loads in vegetation samples and nestling feathers was reported by Dauwe et al. (2004a) for an intensely studied pollution gradient. In an earlier study, Dauwe et al. (2000) found significantly higher lead concentrations in great tit (4.83 µg/g) and blue tit (3.68 µg/g) nestling feathers collected from a more polluted site. In contrast, zinc concentration was significantly higher at the reference site. Moreover, Dauwe et al. (2000) showed that in excrements, all metals, including arsenic, cadmium and copper, had higher concentrations in the more polluted sites. The investigators suggested that the difference between the amounts of metals in excrements and in feathers of great and blue tit nestlings can be explained by the operation of homeostatic mechanisms (excretion of larger amounts of metals through excrements).

Compared with other studies, we found relatively high metal concentrations in feathers of blue tit and great tit nestlings. However, direct comparisons of the results may not be justified because of methodological differences between studies. Specifically, in this study, we sampled secondary flight feathers, whereas Dauwe et al. (2000, 2004a) used the outermost tail feathers. Different types of feathers develop at different rates, and the stage of feather development may influence metal concentration. Dauwe et al. (2000) collected tail feathers that were not fully grown and consisted mostly of the shaft, which, according to Goede and de Bruin (1984), contain lower metal levels than the vanes. Burger and Gochfeld (1992) found significantly lower levels of lead and mercury in growing feathers than in fully formed feathers. Because the secondary flight feathers used in this study were more advanced in development than tail feathers and therefore might have accumulated more heavy metals, they may reflect pollution in a more adequate manner.

We found that mean metal concentrations in feathers of nestlings of both tit species differed between years. Concentrations of lead and zinc for both species and the concentration of cadmium for blue tit nestlings showed a similar pattern of variation with the means for 2007 being lowest. This suggests that some common factors may be responsible for this pattern. A possible factor, highly variable among the years of the study, is rainfall amount during the nesting stage of tits. A brood timing-specific sum of rainfall was also lowest in 2007, which could potentially contribute to the variation in feather metal loads. Taking into account that heavy metals are relatively easily biomagnified, it seems that it is appropriate to analyze how rainfalls impact plants, which are first-level trophic chain organisms. A few studies showed the influence of rainfalls on variations in heavy-metal concentration in vegetation samples. The presence of metals in plants may be an effect of endogenous accumulation (Bingöl et al. 2008), but they may also result from concurring endogenous and exogenous pathways of metal contamination (Ho and Tai 1979; Steinnes 1987; Eriksson et al. 1996; Thöni et al. 2011). Bingöl et al. (2008) reported markedly higher lead and nickel levels in trees *Sophora japonica* L. during rainy seasons. According to Eriksson et al. (1996), cadmium concentrations accumulated in crops were positively correlated with precipitation. The studies performed in Norway also emphasized the relationship of rainfalls and heavy-metal amounts in the environment. Steinnes (1987) noted that greater heavy-metal concentrations were found in vegetation samples collected from areas characterized by a high precipitation level. A similar conclusion can be drawn from the study performed by Thöni et al. (2011), who showed that amounts of metals accumulated in mosses coincided with rainfalls. The investigators proved that lower rainfalls resulted in a lower input of heavy metals. Therefore, we also suggest that in our study sites, heavy metals can be introduced to the environment in the quantities dependent on the amounts of rainfall.

Furthermore, the influence of rainfall may indirectly affect the uptake of metals. According to Leech and Crick (2007) heavy rain decreases the number of available invertebrates by washing them down from the vegetation. Such conditions may cause a limitation of food for canopy-feeders and force them to forage on the ground, and this, as has been shown earlier, may result in supplying offspring with more contaminated food. We suppose that this can explain why, in general, we recorded higher values of analyzed trace elements in years with higher precipitation level. However, we are aware of the need to conduct further research to confirm and refine these findings.

The interyear variation in metal concentrations in birds, particularly in tit species, has not been studied intensely. Janssens et al. (2003) examined the influence of exposure to heavy metals on the condition and health of great tit nestlings in three consecutive breeding seasons, but they did not directly analyze interannual variation. However, it is possible to use their published data to assess interyear differences (Eens et al. 1999; Dauwe et al. 2002, 2004a; Janssens et al. 2002). Dauwe et al. (2004a) showed lower values of lead, higher concentrations of cadmium, and slightly higher loads of zinc in the comparison with the results for 1999 presented by Janssens et al. (2002). The comparison of results obtained by Eens et al. (1999) and by Dauwe et al. (2002) shows that concentrations of lead and cadmium in feathers of adult blue tits also differed between years at the same study sites. Those differences can be explained by changes in the functioning of industrial facilities in the study areas. Similar conclusions can be drawn from the study on magpies *Pica pica* by Dmowski (1997), who noted a marked decrease over the years of lead and cadmium levels in magpie feathers after the activity of steelworks were limited at the turn of the 1990s. Thus, it appears that long-term variation in heavy-metal accumulation is related to changes in the pressure of anthropogenic factors. Closing large industrial factories or decreasing contamination extorted by introducing new, rigorous norms and regulations may significantly affect the overall level of heavy metals in the environment and also their amounts accumulated by birds (Dmowski 1997; Dauwe et al. 2002; Janssens et al. 2003).

In conclusion, our results suggest that intersite variation in heavy-metal concentrations in nestlings of the great and blue tit, although not significant in all cases, reflect the level of contamination occurring at the study sites. We showed that concentrations of trace elements may also change between subsequent years. We suppose that rainfall may be considered an important factor determining the mobility of heavy metals in the environment and, thus, a factor enhancing their bioavailability for organisms, but this needs further research.
